# Acidosis in arterial blood gas testing is associated with clinical outcomes after endovascular thrombectomy

**DOI:** 10.3389/fneur.2022.1077043

**Published:** 2022-12-21

**Authors:** Rui Shao, Lei Liu, Juan Xu, Pengpeng Lan, Guiping Wu, Hongfeng Shi, Ruili Li, Yingle Zhuang, Shanshan Han, Yan Li, Ping Zhao, Min Xu, Ziren Tang

**Affiliations:** ^1^Department of Emergency Medicine, Beijing Chaoyang Hospital, Capital Medical University, Beijing, China; ^2^Department of Internal Medicine, The Affiliated Hospital of China University of Petroleum (East China), Qingdao, China; ^3^Neurological Intensive Care Department, Shengli Oilfield Central Hospital, Dongying City, China

**Keywords:** acidosis, arterial blood gas, hemorrhage, reperfusion, stroke, thrombectomy

## Abstract

**Background:**

Despite recanalization, some of the patients undergoing endovascular thrombectomy (EVT) still suffer from unfavorable outcomes. Patients with poor prognoses are often accompanied by acidosis in arterial blood gas (ABG) testing. We, therefore, explored the ABG testing results in the early phase of recanalization and analyzed their association with poor prognosis.

**Patients and methods:**

We identified all patients with ischemic stroke and successful endovascular recanalization for anterior circulation vessel occlusion between June 2019 and May 2022. ABG testing was performed in all patients within 0–30 min and 8 h after endovascular therapy. We investigated the relationship between the ABG testing results with symptomatic intracerebral hemorrhage (sICH), hemicraniectomy, and mortality.

**Results:**

A total of 123 patients with stroke after endovascular thrombectomy were analyzed. Of those, eight (6.5%) patients had postinterventional sICH. Acidosis was associated with sICH. Decreased ${\text{HCO}}_{3}^{-}$ levels and ${\text{HCO}}_{3}^{-}$ levels at 8 h after EVT were independently related to a higher risk of sICH. Twelve (9.8%) patients underwent hemicraniectomy for postischemic malignant edema and similar results were found for hemicraniectomy. Increased lactate at 8 h after EVT and decreased ${\text{HCO}}_{3}^{-}$ levels at 8 h after EVT were closely associated with hemicraniectomy. Twenty-two (17.9%) patients died within 3 months. Decreased ${\text{HCO}}_{3}^{-}$ levels were independently related to mortality, as were decreased pH levels at 8 h after EVT and decreased ${\text{HCO}}_{3}^{-}$ levels at 8 h after EVT.

**Conclusion:**

Acidosis is associated with clinical outcomes after endovascular therapy and may help to select patients with poor prognosis in the acute early phase of recanalization.

## Introduction

Endovascular thrombectomy is the optimal treatment option in patients with large vessel occlusion acute ischemic stroke (1). Although 90% of patients achieved successful recanalization and the procedure is generally relatively safe, some of the patients still suffer from unfavorable outcomes at 90 days (2–4). Reperfusion injury and hemorrhagic transformation, which are mainly attributed to impaired cerebral blood flow autoregulation and disrupted blood–brain barrier (BBB), have been identified as important complications in the acute phase of recanalization (5–7).

Early identification of potential candidates who may suffer from neurological deterioration should be performed for a timely experimental treatment to improve the outcome of patients with reperfusion therapies. While timely neurological assessment is unavailable in patients who received general anesthesia or mild sedation during endovascular reperfusion therapies. Transcranial duplex (TCD) sonography or transcranial color-coded sonography (TCCS) may be one of the early hemodynamic predictors of outcome, while there are numerous confounding factors affecting middle cerebral artery (MCA) flow velocity. It may be difficult to reverse the prognosis when the intracranial lesion is detected on cerebral computed tomography (CT) or MRI 24 h routinely after the endovascular procedure or in the event of clinical deterioration.

In clinical practice, we often find that patients with poor prognoses have decreased bicarbonate (${\text{HCO}}_{3}^{-}$) levels in arterial blood gas (ABG) testing or even have metabolic acidosis or elevated lactate in the early phase of recanalization. Patients with cerebral infarction are less likely to suffer from hemodynamic disorders in the early stages, so changes in the acid–base balance in peripheral blood may be related to cerebral metabolism. There have been few studies exploring the relationship between ABG testing results and poor prognosis in patients with reperfusion therapies. We, therefore, explored the ABG testing results in the early phase of recanalization and analyzed their association with poor prognosis in patients with successful revascularization.

## Patients and methods

### Patients and study design

Between June 2019 and May 2022, we identified consecutive patients with anterior circulation large vessel occlusion who had undergone endovascular recanalization therapy at Shengli Oilfield Central Hospital, an urban university tertiary hospital and national advanced stroke center. In the treatment protocol for patients with endovascular therapy in our hospital, patients were intubated under general anesthesia by dedicated neuroanesthesiologists in the operating room, followed by endovascular treatment. Then, patients were administrated in the postinterventional period at our neurointensive care unit. Patients would receive mild sedation and close blood pressure monitoring, and they would be evaluated for extubation 12–24 h after endovascular therapy. Patients were included if they underwent endovascular thrombectomy with successful reperfusion. Successful reperfusion was defined as modified thrombolysis in cerebral ischemia (mTICI) ≥ 2b. Additional inclusion criteria were admission Alberta Stroke Program Early CT Score (ASPECTS) ≥ 6. Patients with hemodynamic instability, hepatic, or renal dysfunction on admission will be excluded from the study. Patients with secondary metabolic abnormalities, such as diabetic ketoacidosis, mitochondrial disease, respiratory acidosis, respiratory alkalosis, or severe aspiration pneumonia were also excluded. Clinical characteristics, including demographic characteristics, past medical history, drug usage, the National Institutes of Health Stroke Scale (NIHSS) scores, intravenous thrombolysis, occlusion location, onset-to-groin puncture time, final mTICI grades, postoperative blood pressure, stroke complication, and outcome were collected. All patients were treated according to current stroke guidelines (1). The Hospital Institutional Review Board and the Ethics Committee of the Shengli Oilfield Central Hospital approved the study. Analysis of patient data was performed in accordance with the Declaration of Helsinki. All patients performed cerebral CT or MRI 24 h after thrombectomy with successful revascularization, and additional CT was also performed in case of clinical deterioration. The primary endpoints were symptomatic intracranial hemorrhage (sICH), all-cause 90-day mortality, and whether or not hemicraniectomy was performed during hospitalization. sICH was defined as any apparent extravascular blood in the brain or within the cranium that was associated with clinical deterioration, as defined by an increase of >2 points in one category or >4 points in total on the NIHSS (6, 8). Functional status was assessed 3 months after stroke onset using the modified Rankin Scale (mRS). The mRS was collected through telephone interviews.

### TCD and ABG testing

Transcranial duplex sonography (devices: Delica EMS-9PB, Delicate Manufacturer, Shenzhen, China) and ABG testing (devices: GEM premier 4000, Werfen Co. Barcelona, Spain) were performed in all patients within 0–30 min and 8 h after endovascular therapy as bedside assessment at neurointensive care unit. Absolute values for mean blood flow (MBF) velocities and pulsatility indices (PI) were analyzed in the treated (ipsilateral) MCA. pH, actual bicarbonate (${\text{HCO}}_{3}^{-}$) levels, and lactate levels in ABG testing from radial artery were recorded and analyzed.

### Statistical analysis

The baseline characteristics were described as frequencies, percentages, median, and interquartile ranges. Comparisons between groups were made using the Mann–Whitney *U*-test for continuous variables. Receiver operating characteristic (ROC) curves and the area under the ROC curve (AUC) were conducted. Multivariable logistic regression analyses were performed for each group to determine factors that could be considered independent predictors of clinical outcomes. Clinically relevant variables and those showing *P* < 0.05 in univariate analysis were included in the multivariate model. The model was determined by the method of ENTER (default with the menu system). *P* < 0.05 was considered to be statistically significant. All statistical analyses were carried out using SPSS 19.0 (SPSS Inc., Chicago, IL) and GraphPad Prism 6 (GraphPad, La Jolla, CA).

## Results

### Characteristics of enrolled subjects

**Figure 1:**
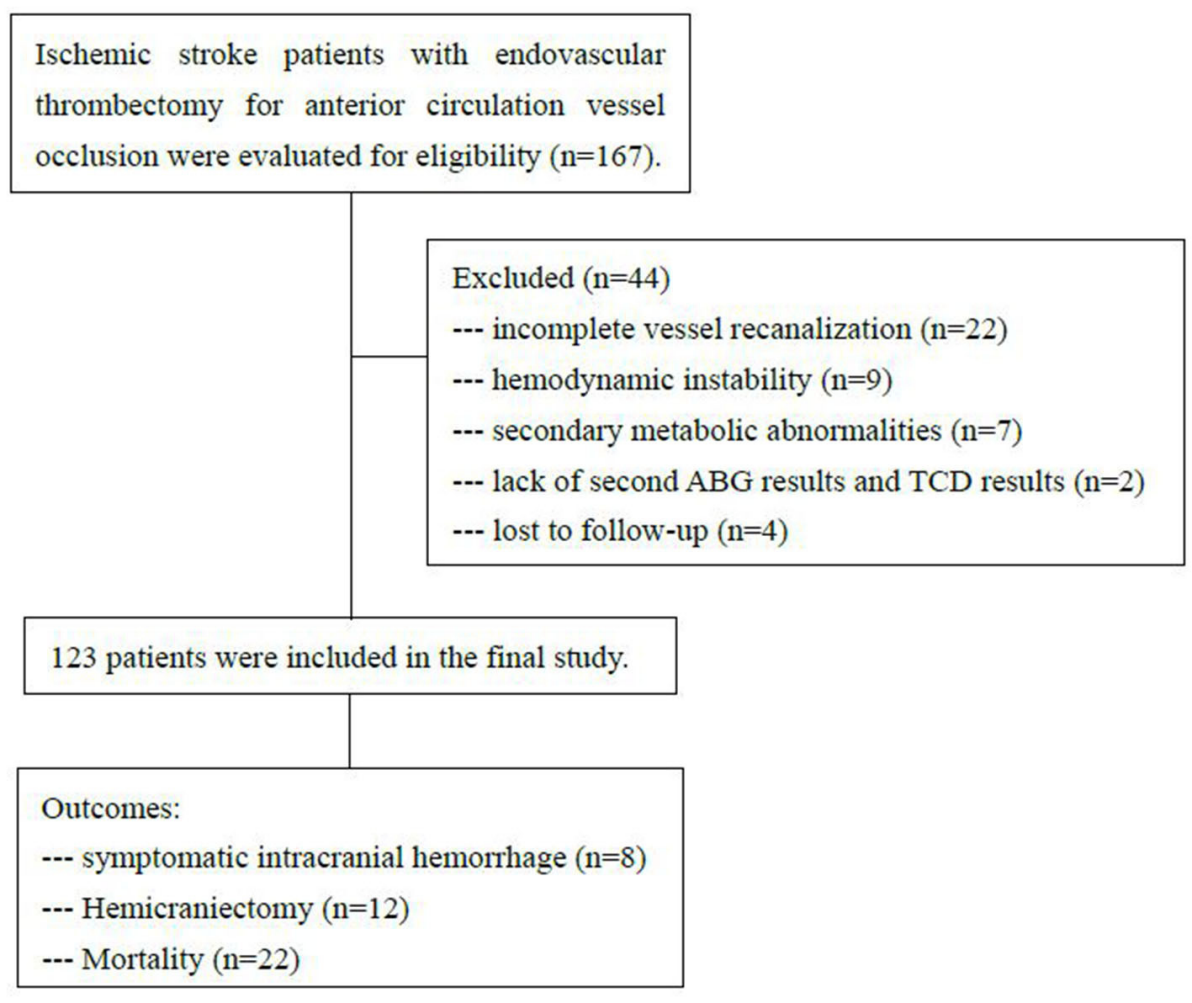
Research flow chart. ABG, arterial blood gas (ABG); TCD, transcranial duplex.

During the investigational period, 167 patients received endovascular recanalization therapy with anterior circulation large vessel occlusion. A total of 44 patients were excluded. Twenty-two patients with incomplete vessel recanalization (TICI 0-2a) and nine patients with hemodynamic instability were excluded. Seven patients with secondary metabolic abnormalities were excluded, including one patient with mitochondrial disease, four patients with severe aspiration pneumonia, and two patients with diabetic ketoacidosis. Two patients were excluded due to the lack of second ABG results and TCD results, and these patients had gone to the operating room for hemicraniectomy within 8 h. Four patients were lost to follow-up. A total of 123 patients were enrolled in the study. The flow chart is illustrated in Figure 1.

Table 1 summarizes the baseline characteristics and outcomes of the study cohort. Fifty-three (43%) patients were women, with a median age of 66 years [interquartile range (IQR), 63–74], median NIHSS score of 17 (IQR, 13–20), and median ASPECT score of 9 (IQR, 8–10). Distribution of initial arterial occlusion location was as follows: Terminal intracranial internal carotid artery 23 (18%), M1-middle cerebral artery 56 (46%), M2-middle cerebral artery 28 (23%), and tandem occlusion 16 (13%). Sixty-six patients (54%) received intravenous thrombolysis before endovascular thrombectomy (EVT). After a median time of 256 min (221–312) from symptoms onset to groin puncture, 80 (65%) patients achieved a final mTICI 3 and 43 (35%) achieved mTICI 2b.

**Table 1:** Baseline characteristics and outcomes of the study cohort.

**Variable**	
**Demographics**	
Age, year, median (IQR)	66 (63–74)
Female sex, *n* (%)	53 (43%)
Diabetes mellitus, *n* (%)	41 (33%)
Hypertension, *n* (%)	68 (55%)
Hyperlipidemia, *n* (%)	57 (46%)
Atrial fibrillation, *n* (%)	42 (34%)
Smoker on admission, *n* (%)	34 (28%)
Anticoagulation use, *n* (%)	10 (8%)
Antiplatelet use, *n* (%)	39 (32%)
**Clinical features**	
NIHSS, median (IQR)	17 (13–20)
ASPECTS, median (IQR)	9 (8–10)
MBP, mmHg, median (IQR)	79 (73–86)
**Occlusion site**, ***n*** **(%)**	
TICA	23(18%)
M1-MCA	56 (46%)
M2-MCA	28 (23%)
Tandem occlusion	16 (13%)
**Procedural features**	
IV tPA, *n* (%)	66 (54%)
Onset-to-Groin, min, median (IQR)	256 (221–312)
Final mTICI 3, *n* (%)	80 (65%)
Final mTICI 2b, *n* (%)	43 (35%)
**Outcomes**	
mRS score ≤ 2 at 90 days, *n* (%)	58 (47%)
sICH, *n* (%)	8 (6.5%)
Hemicraniectomy, *n* (%)	12 (9.8%)
Mortality, *n* (%)	22 (17.9%)

ASPECTS, alberta stroke program early CT score; IQR, interquartile range; IV tPA, intravenous tissue-type plasminogen activator; MBP, mean blood pressure; MCA, middle cerebral artery; mRS, modified rankin scale; mTICI, modified thrombolysis in cerebral ischemia score; NIHSS, national institutes of health stroke scale; sICH, symptomatic intracranial hemorrhage; TICA, terminal artery carotid artery.

### Patients with symptomatic intracranial hemorrhage

**Figure 2:**
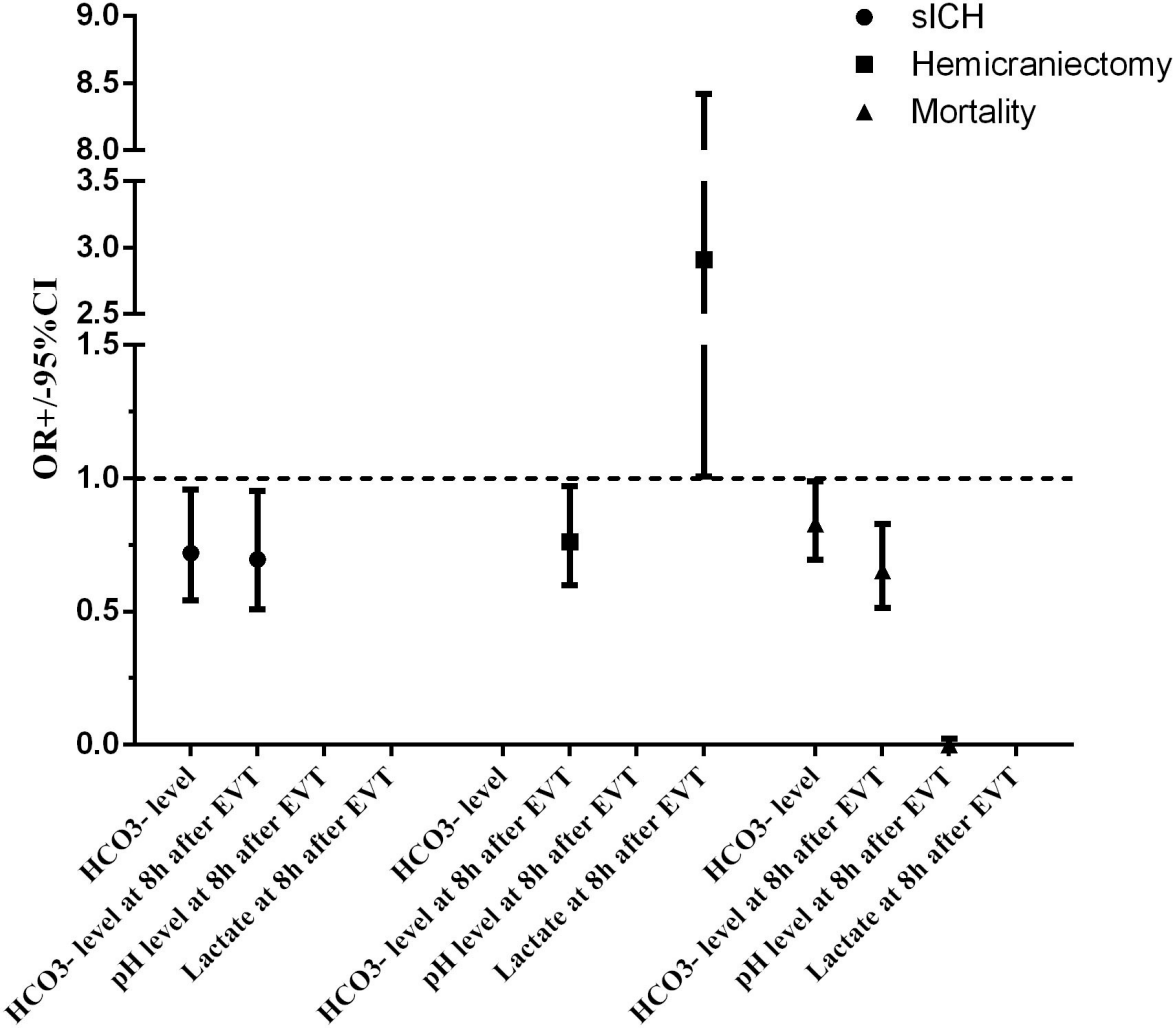
Adjusted odds ratio (OR, midpoints) and 95% CIs (error bars) derived from multivariable analysis for clinical outcomes of patients with endovascular thrombectomy. ASPECTS, Alberta stroke program early CT score; CI, confidence interval; EVT, endovascular thrombectomy; HCO^3−^, bicarbonate; NIHSS, national institutes of health stroke scale; OR, odds ratio; sICH, symptomatic intracranial hemorrhage.

**Figure 3:**
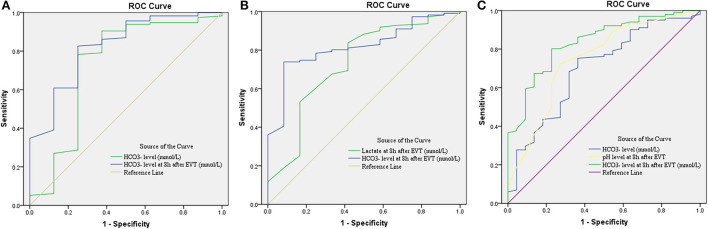
Receiver operating characteristic (ROC) curve for predicting sICH **(A)**, hemicraniectomy **(B)**, and mortality **(C)** in patients with endovascular thrombectomy. CI, confidence interval; EVT, endovascular treatment; ${\text{HCO}}_{3}^{-}$, bicarbonate; sICH, symptomatic intracranial hemorrhage.

A total of eight (6.5%) patients developed sICH after thrombectomy in this study. Patients with sICH had significantly lower ${\text{HCO}}_{3}^{-}$ levels (*P* = 0.028). pH levels at 8 h after EVT and ${\text{HCO}}_{3}^{-}$ levels at 8 h after EVT were also significantly reduced in patients with sICH (*P* = 0.006 and *P* = 0.001). Although there was a tendency for MBF velocity and PI to increase in patients with sICH, only PI at 8 h after EVT was significantly increased (*P* = 0.037). The detailed results are shown in Table 2. Univariate and multivariate logistic regression were used to identify independent predictors associated with sICH. After the univariate analysis, age, NIHSS score, ASPECTS, and onset-to-groin puncture time were included in the final model. Using the multivariate logistic regression analysis, decreased ${\text{HCO}}_{3}^{-}$ levels were independently associated with sICH [odds ratio (OR) = 0.720, 95% confidence interval (CI) 0.541–0.958, *P* = 0.024], as were decreased ${\text{HCO}}_{3}^{-}$ levels at 8 h after EVT (OR = 0.696, 95% CI: 0.509–0.952, *P* = 0.023). The detailed results are illustrated in Figure 2 and Table 3. According to the ROC curve, the AUC of the ${\text{HCO}}_{3}^{-}$ levels for predicting sICH was 0.730 (95% CI: 0.496–0.964, *P* = 0.030), and ${\text{HCO}}_{3}^{-}$ levels at 8 h after EVT was 0.824 (95% CI: 0.674–0.975, *P* = 0.002). The detailed results are demonstrated in Figure 3A.

**Table 2:** Comparison between favorable and poor prognosis according to the outcome: Symptomatic intracranial hemorrhage.

**Variables**	**No sICH**	**sICH**	***P*-value**
MBF velocity after EVT (cm/s)	71 (57–85)	81 (71–100)	0.126
PI after EVT	1.04 (0.84–1.14)	0.94 (0.88–1.29)	0.734
MBF velocity at 8 h after EVT (cm/s)	71 (59–86)	83 (71–99)	0.079
PI at 8 h after EVT	1.05 (0.90–1.18)	1.17 (1.08–1.30)	0.037
pH level	7.36 (7.34–7.40)	7.31 (7.27–7.41)	0.215
Lactate level (mmol/L)	1.1 (0.9–1.9)	1.5 (1.1–2.1)	0.113
${\text{HCO}}_{3}^{-}$ level (mmol/L)	22.6 (20.5–25.0)	17.6 (15.6–23.5)	0.028
pH level at 8 h after EVT	7.39 (7.36–7.41)	7.34 (7.29–7.37)	0.006
Lactate at 8 h after EVT (mmol/L)	1.3 (1.0–1.8)	1.3 (0.9–2.5)	0.471
${\text{HCO}}_{3}^{-}$ level at 8h after EVT (mmol/L)	24.2 (21.0–25.6)	18.8 (16.1–22.4)	0.001

Data are shown as median and interquartile range unless otherwise indicated.

EVT, endovascular thrombectomy; ${\text{HCO}}_{3}^{-}$, bicarbonate; MBF, mean blood flow; PI, pulsatility indices; sICH, symptomatic intracranial hemorrhage.

**Table 3:** Logistic regression analysis of independent factors for prognosis in patients with endovascular reperfusion therapies.

**Variable**	***B***	**SE**	**Wald**	***P*-value**	**OR**	**95 % confidence interval for EXP(B)**
${\text{HCO}}_{3}^{-}$ level*	−0.329	0.146	5.085	0.024	0.720	0.541–0.958
${\text{HCO}}_{3}^{-}$ level at 8 h after EVT*	−0.362	0.160	5.134	0.023	0.696	0.509–0.952
Lactate at 8 h after EVT^#^	1.068	0.542	3.885	0.049	2.910	1.006–8.419
${\text{HCO}}_{3}^{-}$ level at 8 h after EVT^#^	−0.271	0.123	4.889	0.027	0.762	0.599–0.970
${\text{HCO}}_{3}^{-}$ level^&^	−0.188	0.090	4.353	0.037	0.829	0.695–0.989
pH level at 8 h after EVT^&^	−19.364	7.923	5.973	0.015	0	0–0.022
${\text{HCO}}_{3}^{-}$ level at 8 h after EVT^&^	−0.427	0.122	12.290	< 0.001	0.652	0.514–0.828

^*^Multivariable logistic regression analyses were performed for symptomatic intracranial hemorrhage. ^#^Multivariable logistic regression analyses were performed for hemicraniectomy, adjusting for hyperlipidemia. ^&^Multivariable logistic regression analyses were performed for mortality, adjusting for diabetes mellitus, NIHSS score, ASPECTS, tandem occlusion, and onset-to-groin puncture time.

ASPECTS, alberta stroke program early CT score; EVT, endovascular thrombectomy; ${\text{HCO}}_{3}^{-}$, bicarbonate; NIHSS, national institutes of health stroke scale.

### Patients with hemicraniectomy

A total of 12 (9.8%) patients underwent postischemic malignant edema. Hemicraniectomy was regarded as a surrogate for malignant edema, and we investigated the relationship between relevant variables and hemicraniectomy. Patients with hemicraniectomy had significantly increased MBF velocity at 8 h after EVT (*P* = 0.048). Lactate levels and lactate at 8 h after EVT were obviously higher in patients with hemicraniectomy (*P* = 0.027 and *P* = 0.007). pH levels, ${\text{HCO}}_{3}^{-}$ levels, pH levels at 8 h after EVT, and ${\text{HCO}}_{3}^{-}$ levels at 8 h after EVT were significantly reduced in the hemicraniectomy group (all *P* < 0.05). The detailed results are shown in Table 4. After the univariate analysis, hyperlipidemia, atrial fibrillation, NIHSS score, ASPECTS, tandem occlusion, and onset-to-groin puncture time were included in the multivariable analysis. Increased lactate at 8 h after EVT was an independent predictor of hemicraniectomy (OR = 2.910, 95% CI: 1.006–8.419, *P* = 0.049), as was decreased ${\text{HCO}}_{3}^{-}$ levels at 8 h after EVT (OR = 0.762, 95% CI: 0.599–0.970, *P* = 0.027). The detailed results are shown in Figure 2 and Table 3. The AUC of lactate at 8 h after EVT for predicting hemicraniectomy was 0.731 (95% CI: 0.568–0.894, *P* = 0.009). ${\text{HCO}}_{3}^{-}$ levels at 8 h after EVT were 0.818 (95% CI: 0.716–0.920, *P* < 0.001). The detailed results are illustrated in Figure 3B.

**Table 4:** Comparison between favorable and poor prognosis according to the outcome: Hemicraniectomy performed during hospitalization.

**Variables**	**No hemicraniectomy**	**Hemicraniectomy**	***P*-value**
MBF velocity after EVT (cm/s)	71 (56–85)	77 (72–91)	0.077
PI after EVT	1.04 (0.84–1.14)	1.06 (0.96–1.20)	0.215
MBF velocity at 8 h after EVT (cm/s)	71 (58–87)	80 (77–90)	0.048
PI at 8 h after EVT	1.05 (0.90–1.17)	1.14 (1.03–1.25)	0.079
pH level	7.37 (7.34–7.40)	7.31 (7.29–7.36)	0.014
Lactate level (mmol/L)	1.1 (0.9–1.9)	1.8 (1.2–2.1)	0.027
${\text{HCO}}_{3}^{-}$ level (mmol/L)	22.9 (20.6–25.1)	19.4 (16.1–20.3)	< 0.001
pH level at 8 h after EVT	7.39 (7.36–7.42)	7.35 (7.31–7.37)	< 0.001
Lactate at 8 h after EVT (mmol/L)	1.2 (0.90–1.7)	2.2 (1.3–2.5)	0.007
${\text{HCO}}_{3}^{-}$ level at 8h after EVT (mmol/L)	24.2 (21.1–25.6)	20.2 (17.2–21.2)	< 0.001

Data are shown as median and interquartile range unless otherwise indicated.

EVT, endovascular thrombectomy; ${\text{HCO}}_{3}^{-}$, bicarbonate; MBF, mean blood flow; PI, pulsatility indices.

### Patients with mortality

Twenty-two (17.9%) patients died within 3 months (mRS score = 6) in this study. The mortality group had higher PI at 8 h after EVT (*P* = 0.010) and lactate at 8 h after EVT (*P* = 0.041). ${\text{HCO}}_{3}^{-}$ levels, pH levels at 8 h after EVT, and ${\text{HCO}}_{3}^{-}$ levels at 8 h after EVT were significantly lower in the mortality group (Table 5). Adjusting diabetes mellitus, NIHSS score, ASPECTS, tandem occlusion, onset-to-groin puncture time, and decreased ${\text{HCO}}_{3}^{-}$ levels were independent predictors of mortality within 3 months in the multivariable analysis (OR = 0.829, 95% CI: 0.695–0.989, *P* = 0.037), as were decreased pH levels at 8 h after EVT (OR = 0, 95% CI: 0–0.022, *P* = 0.015) and decreased ${\text{HCO}}_{3}^{-}$ levels at 8 h after EVT (OR = 0.652, 95% CI: 0.514–0.828, *P* < 0.001). The detailed results are presented in Figure 2 and Table 3. The AUC of ${\text{HCO}}_{3}^{-}$ levels for predicting mortality was 0.700 (95% CI: 0.578–0.821, *P* = 0.003). pH levels at 8 h after EVT were 0.747 (95% CI: 0.629–0.866, *P* < 0.001), and ${\text{HCO}}_{3}^{-}$ levels at 8 h after EVT were 0.837 (95% CI: 0.751–0.923, *P* < 0.001). The detailed results are shown in Figure 3C.

**Table 5:** Comparison between favorable and poor prognosis according to the outcome: Mortality.

**Variables**	**mRS < 6**	**mRS = 6**	***P*-value**
MBF velocity after EVT (cm/s)	71 (55–85)	76 (71–92)	0.055
PI after EVT	1.04 (0.88–1.13)	0.97 (0.81–1.18)	0.815
MBF velocity at 8 h after EVT (cm/s)	71 (58–86)	78 (70–93)	0.151
PI at 8 h after EVT	1.05 (0.90–1.13)	1.20 (1.01–1.28)	0.010
pH level	7.37 (7.34–7.40)	7.34 (7.30–7.39)	0.058
Lactate level (mmol/L)	1.1 (0.9–1.9)	1.2 (1.0–2.0)	0.161
${\text{HCO}}_{3}^{-}$ level (mmol/L)	22.9 (20.7–25.2)	20.5 (16.6–23.6)	0.003
pH level at 8 h after EVT	7.39 (7.36–7.42)	7.35 (7.31–7.39)	< 0.001
Lactate at 8 h after EVT (mmol/L)	1.2 (0.9–1.7)	1.4 (1.2–2.1)	0.041
${\text{HCO}}_{3}^{-}$ level at 8 h after EVT (mmol/L)	24.2 (22.2–25.9)	19.6 (17.9–21.5)	< 0.001

Data are shown as median and interquartile range unless otherwise indicated.

EVT, endovascular thrombectomy; ${\text{HCO}}_{3}^{-}$, bicarbonate; MBF, mean blood flow; mRS, modified Rankin Scale; PI, pulsatility indices.

## Discussion

Despite revascularization after thrombectomy, a relevant proportion of patients with large vessel occlusion acute ischemic stroke do not achieve a favorable prognosis. Early identification of patients with possible neurological deterioration may be critical to improving prognosis. Our study demonstrated that ABG testing results were closely related to clinical outcomes after EVT. Our main finding was that acidosis in arterial blood gas testing was strongly associated with poor prognosis. Decreased ${\text{HCO}}_{3}^{-}$ levels at 8 h after EVT were independently related to higher odds of sICH, hemicraniectomy, and mortality. Decreased ${\text{HCO}}_{3}^{-}$ levels, decreased pH at 8 h after EVT, and elevated lactate levels at 8 h after EVT were also presented in patients with poor clinical outcomes. To the best of our knowledge, this was the first study to investigate the relationship between ABG testing results and clinical outcomes after successful recanalization. Our findings may be useful for screening patients with possible neurological deterioration in the acute early phase of recanalization. ABG could detect exacerbations earlier than routine TCD, CT, or MRI.

After successful recanalization, a series of complex pathophysiological changes occur in the cerebral tissue, which may involve the intricate interplay of mitochondrial dysfunction, overload of calcium, excitotoxicity, free radical-mediated toxicity, endothelial pathology, and edema formation (9–13). In another view, recanalization after EVT does not always lead to downstream reperfusion, a state that has been known as the no-reflow phenomenon, which may be due to capillary compression and luminal narrowing in cerebral circulation (14–16). These pathophysiologic derangements may result in cerebral microcirculation disorders. The larger the infarct size, the more severe the microcirculation disorders may be. Impaired microcirculation could lead to excessive acid production, increased toxic substances, and elevated lactic acid. Due to exacerbation of BBB disruption after reperfusion, these acid productions are released into peripheral blood (17). Dysfunctional cerebral blood flow autoregulation after thrombectomy aggravates these processes (18).

The existence of a buffer system in the body will maintain pH in the normal range during the initial phase. However, ${\text{HCO}}_{3}^{-}$ as the main component of the biological buffer may change at the onset. Under normal conditions, lactate is continually being produced and metabolized, maintaining dynamic balance. When lactate production predominates, lactate levels rise. Impaired microcirculation can lead not only to elevated lactate levels but also to excessive production of other acids. For example, in the area of cerebral infarction, the Na^+^/H^+^ exchange isoform 1 (NHE1) function is augmented (19). As a result, Na^+^ enters the cells in return for H^+^ extrusion, which may aggravate acidosis (20). Thus, bicarbonate, as the main component of biological buffer, is a more comprehensive and earlier indicator of cerebral metabolism, whereas lactate is only a major part of metabolic acidosis. Acidosis (drop in pH) may present when the buffering system fails to compensate. Excluding causes such as systemic hypoperfusion and other causes of secondary metabolic abnormalities, pH, lactate levels, and ${\text{HCO}}_{3}^{-}$ levels may be optimal candidates for embodying a cerebral metabolic state, which may indicate the severity of cerebral postischemic reperfusion injury.

Previous studies have explored cerebral metabolism in cerebral ischemia under experimental and clinical conditions by using microdialysis catheters. In animal experiments, the biochemical pattern of cerebral tissue during ischemia is characterized by a marked increase in cerebral lactate (21, 22). Elevated intracerebral lactate and glutamate were also demonstrated in patients with MCA infarcts and malignant brain swelling (23, 24). In addition, the Na^+^/H^+^ exchange is augmented and tissue pCO_2_ rises during ischemia, which may also contribute to cerebral tissue acidosis (19, 25). Acidic amino acids, lactate, and other acidic metabolites were released into the peripheral blood through the damaged BBB, and these products resulted in altering biological buffers, even metabolic acidosis.

The previous study has shown that bedside TCD indicated a significantly higher MBF velocity in the recanalized MCA in patients who experienced ICH following recanalization (26). Although there was a tendency for MBF velocity to increase in patients with sICH in our study, the MBF velocity was not a statistically significant difference between the two groups. The difference between our results and the previous study could be related to the fact that TCD in the present study was performed within 0–30 min and 8 h after EVT, Markus et al. performed a TCD examination within 24 h after EVT. However, the two studies were similar in that MBF velocity was not an independent predictor of sICH following recanalization. Therefore, MBF velocity may not be an optimal biomarker to identify patients with possible neurological deterioration in the acute early phase of recanalization.

Cerebral metabolism is accompanied by complex pathophysiological changes in patients with large vessel occlusion acute ischemic stroke. After successful recanalization, cerebral metabolism may suffer from aggravation due to postischemic reperfusion injury. There are more published articles using bedside TCD to explore cerebral blood flow in patients with cerebral infarction (27, 28). However, it is surprising that so scant attention has been given to bedside cerebral metabolic markers. Cerebral metabolic indicators may contribute appropriate information on cerebral impairment much earlier. In this context, ABG testing as an efficient bedside screening method is certainly more readily available than other examinations and provides more information about prognosis.

In this study, we observed that decreased ${\text{HCO}}_{3}^{-}$ levels at 8 h after EVT were a reliable predictor of clinical outcome in patients with successful revascularization. In the case of conventional treatment after recanalization, decreased ${\text{HCO}}_{3}^{-}$ levels at 8 h after EVT indicated that acid production from cerebral metabolism is still increasing, implying deteriorating cerebral function. This study demonstrated that increased lactate at 8 h after EVT was closely associated with hemicraniectomy, which was regarded as a surrogate for malignant edema. Increased lactate levels may result from microcirculatory disorders provoking secondary brain impairment. Due to severe deterioration of cerebral function and excessive acid production release, patients who died may be accompanied by a decrease in pH levels after endovascular therapy.

Some limitations need to be considered in this study. First, all newly admitted patients had their ABG testing routinely collected within 30 min and reviewed 8 h later in our intensive care unit. BBB permeability was increased in the ischemic hemisphere 1 h after reperfusion (29), and whether an earlier review of the ABG testing could provide useful information about the prognosis needs to be further explored. Second, although we included only patients with ASPECTS ≥ 6, we could not obtain the final infarct volume. The correlation of final infarct volume with ABG testing results may provide more useful information. Third, the sample sizes were relatively small, this was a single-center study, and more patients need to be included to confirm these findings. Fourth, a lot of patients with malignant cerebral edema may not choose to undergo hemicraniectomy due to age, hemispheric laterality, and other reasons. But the prognosis of these patients may be worsened and eventually be classified in the death group.

## Conclusion

In the present study, we have indicated that post-EVT ABG testing might be an effective bedside method for assessing prognosis in patients with large vessel occlusion acute ischemic stroke. Acidosis in arterial blood gas testing is associated with clinical outcomes after endovascular therapy and may help to select patients with poor prognosis in the acute early phase of recanalization.

## Data availability statement

The original contributions presented in the study are included in the article/supplementary material, further inquiries can be directed to the corresponding authors.

## Ethics statement

The studies involving human participants were reviewed and approved by the study was approved by the Ethics Committee of the Shengli Oilfield Central Hospital (Approval No. Q/ZXYY-ZY-YWB—LL202251). The patients/participants provided their written informed consent to participate in this study.

## Author contributions

ZT and MX conceived and designed the study. RS, LL, JX, PL, GW, HS, RL, YZ, SH, YL, and PZ acquired the data. RS and LL analyzed the data, which was discussed with ZT and MX. RS and ZT drafted and critically revised the manuscript. All authors gave final approval for manuscript publication and agree to be accountable for all aspects of this work. All authors contributed to the article and approved the submitted version.

## References

[1] Powers WJ Rabinstein AA Ackerson T Adeoye OM Bambakidis NC Becker K. Guidelines for the early management of patients with acute ischemic stroke: 2019 update to the 2018 guidelines for the early management of acute ischemic stroke: a guideline for healthcare professionals from the American Heart Association/American Stroke Association. Stroke. (2019) 50:e344–418. 10.1161/STR.0000000000000211 31662037

[2] Saver JL Goyal M Diener HC; SWIFT PRIME. Investigators. Stent-retriever thrombectomy for stroke. N Engl J Med. (2015) 373:1077. 10.1056/NEJMc1508744 26352820

[3] Berkhemer OA van Zwam WH Dippel DW; MR CLEAN. Investigators. Stent-retriever thrombectomy for stroke. N Engl J Med. (2015) 373:1076. 10.1056/NEJMc1508744 26352821

[4] Yaghi S Eisenberger A Willey JZ. Symptomatic intracerebral hemorrhage in acute ischemic stroke after thrombolysis with intravenous recombinant tissue plasminogen activator: a review of natural history and treatment. JAMA Neurol. (2014) 71:1181–85. 10.1001/jamaneurol.2014.1210 25069522 PMC4592535

[5] Hao Y Yang D Wang H Zi W Zhang M Geng Y. Predictors for symptomatic intracranial hemorrhage after endovascular treatment of acute ischemic stroke. Stroke. (2017) 48:1203–09. 10.1161/STROKEAHA.116.016368 28373302

[6] Wang DT Churilov L Dowling R Mitchell P Yan B. Successful recanalization post endovascular therapy is associated with a decreased risk of intracranial haemorrhage: a retrospective study. BMC Neurol. (2015) 15:185. 10.1186/s12883-015-0442-x 26445968 PMC4597389

[7] Lin YH Liu HM. Update on cerebral hyperperfusion syndrome. J Neurointerv Surg. (2020) 12:788–93. 10.1136/neurintsurg-2019-015621 32414892 PMC7402457

[8] von Kummer R Broderick JP Campbell BC Demchuk A Goyal M Hill MD. The Heidelberg bleeding classification: classification of bleeding events after ischemic stroke and reperfusion therapy. Stroke. (2015) 46:2981–6. 10.1161/STROKEAHA.115.010049 26330447

[9] Fujimura M Morita-Fujimura Y Noshita N Sugawara T Kawase M Chan PH. The cytosolic antioxidant copper/zinc-superoxide dismutase prevents the early release of mitochondrial cytochrome c in ischemic brain after transient focal cerebral ischemia in mice. J Neurosci. (2000) 20:2817–24. 10.1523/JNEUROSCI.20-08-02817.2000 10751433 PMC6772210

[10] Kristal BS Dubinsky JM. Mitochondrial permeability transition in the central nervous system: induction by calcium cycling-dependent and -independent pathways. J Neurochem. (1997) 69:524–38. 10.1046/j.1471-4159.1997.69020524.x 9231710

[11] Achzet LM Davison CJ Shea M Sturgeon I Jackson DA. Oxidative stress underlies the Ischemia/reperfusion-induced internalization and degradation of AMPA receptors. Int J Mol Sci. (2021) 22:717. 10.3390/ijms22020717 33450848 PMC7828337

[12] Sun MS Jin H Sun X Huang S Zhang FL Guo ZN. Free radical damage in ischemia-reperfusion injury: an obstacle in acute ischemic stroke after revascularization therapy. Oxid Med Cell Longev. (2018) 2018:3804979. 10.1155/2018/3804979 29770166 PMC5892600

[13] Bai J Lyden PD. Revisiting cerebral postischemic reperfusion injury: new insights in understanding reperfusion failure, hemorrhage, and edema. Int J Stroke. (2015) 10:143–52. 10.1111/ijs.12434 25598025

[14] Kloner RA. No-reflow phenomenon: maintaining vascular integrity. J Cardiovasc Pharmacol Ther. (2011) 16:244–50. 10.1177/1074248411405990 21821523

[15] Zoppo GJ Schmid-Schönbein GW Mori E Copeland BR Chang CM. Polymorphonuclear leukocytes occlude capillaries following middle cerebral artery occlusion and reperfusion in baboons. Stroke. (1991) 22:1276–83. 10.1161/01.STR.22.10.1276 1926239

[16] Liu S Connor J Peterson S Shuttleworth CW Liu KJ. Direct visualization of trapped erythrocytes in rat brain after focal ischemia and reperfusion. J Cereb Blood Flow Metab. (2002) 22:1222–30. 10.1097/01.wcb.0000037998.34930.83 12368661

[17] Warach S Latour LL. Evidence of reperfusion injury, exacerbated by thrombolytic therapy, in human focal brain ischemia using a novel imaging marker of early blood-brain barrier disruption. Stroke. (2004) 35:2659–61. 10.1161/01.STR.0000144051.32131.09 15472105

[18] Nogueira RC Aries M Minhas JS Petersen N Xiong L Kainerstorfer JM. Review of studies on dynamic cerebral autoregulation in the acute phase of stroke and the relationship with clinical outcome. J Cereb Blood Flow Metab. (2022) 42:430–53. 10.1177/0271678X211045222 34515547 PMC8985432

[19] Manhas N Shi Y Taunton J Sun D. p90 activation contributes to cerebral ischemic damage *via* phosphorylation of Na^+^/H^+^ exchanger isoform 1. J Neurochem. (2010) 114:1476–86. 10.1111/j.1471-4159.2010.06868.x 20557427 PMC2924815

[20] Kintner DB Chen X Currie J Chanana V Ferrazzano P Baba A. Excessive Na^+^/H^+^ exchange in disruption of dendritic Na^+^ and Ca^2+^ homeostasis and mitochondrial dysfunction following *in vitro* ischemia. J Biol Chem. (2010) 285:35155–68. 10.1074/jbc.M110.101212 20817726 PMC2966129

[21] Nordström CH Siesjö BK. Influence of phenobarbital on changes in the metabolites of the energy reserve of the cerebral cortex following complete ischemia. Acta Physiol Scand. (1978) 104:271–80. 10.1111/j.1748-1716.1978.tb06279.x 716981

[22] Nielsen TH Olsen NV Toft P Nordström CH. Cerebral energy metabolism during mitochondrial dysfunction induced by cyanide in piglets. Acta Anaesthesiol Scand. (2013) 57:793–801. 10.1111/aas.12092 23495747

[23] Nielsen TH Ståhl N Schalén W Reinstrup P Toft P Nordström CH. Recirculation usually precedes malignant edema in middle cerebral artery infarcts. Acta Neurol Scand. (2012) 126:404–10. 10.1111/j.1600-0404.2012.01664.x 22494199

[24] Nielsen TH Schalén W Ståhl N Toft P Reinstrup P Nordström CH. Bedside diagnosis of mitochondrial dysfunction after malignant middle cerebral artery infarction. Neurocrit Care. (2014) 21:35–42. 10.1007/s12028-013-9875-5 23860668

[25] von Hanwehr R Smith ML Siesjö BK. Extra- and intracellular pH during near-complete forebrain ischemia in the rat. J Neurochem. (1986) 46:331–9. 10.1111/j.1471-4159.1986.tb12973.x 3079817

[26] Kneihsl M Niederkorn K Deutschmann H Enzinger C Poltrum B Fischer R. Increased middle cerebral artery mean blood flow velocity index after stroke thrombectomy indicates increased risk for intracranial hemorrhage. J Neurointerv Surg. (2018) 10:882–7. 10.1136/neurintsurg-2017-013617 29288194

[27] He YB Su YY Rajah GB Zhang YB Fan LL Liu G. Trans-cranial Doppler predicts early neurologic deterioration in anterior circulation ischemic stroke after successful endovascular treatment. Chin Med J. (2020) 133:1655–61. 10.1097/CM9.0000000000000881 32604178 PMC7401737

[28] Baracchini C Farina F Palmieri A Kulyk C Pieroni A Viaro F. Early hemodynamic predictors of good outcome and reperfusion injury after endovascular treatment. Neurology. (2019) 92:e2774–83. 10.1212/WNL.0000000000007646 31092627

[29] Kahles T Luedike P Endres M Galla HJ Steinmetz H Busse R. NADPH oxidase plays a central role in blood-brain barrier damage in experimental stroke. Stroke. (2007) 38:3000–6. 10.1161/STROKEAHA.107.489765 17916764

